# Influence of supplemented coated-cysteamine on morphology, apoptosis and oxidative stress status of gastrointestinal tract

**DOI:** 10.1186/s12917-019-2076-5

**Published:** 2019-09-13

**Authors:** Hongnan Liu, Miaomiao Bai, Bie Tan, Kang Xu, Rong Yu, Ruilin Huang, Yulong Yin

**Affiliations:** 1 Scientific Observing and Experimental Station of Animal Nutrition and Feed Science in South-Central, Ministry of Agriculture, Hunan Provincial Engineering Research Center for Healthy Breeding of Livestock and Poultry, Key Laboratory of Agro-ecological Processes in Subtropical Region, Institute of Subtropical Agriculture, Chinese Academy of Sciences, 644 Yuanda 2 Road, Changsha, 410125 China; 2 Hangzhou King Techina Technology Company Academician Expert Workstation, Hangzhou King Techina Technology Co., Ltd., Hangzhou, 311107 China; 3 Hunan Co-Innovation Center of Animal Production Safety, CICAPS, Changsha, Hunan 410128 People’s Republic of China; 4 College of Animal Science, South China Agricultural University, Guangzhou, 510642 Guangdong China

**Keywords:** Cysteamine, Coating technology, Gastrointestinal tract, Pigs

## Abstract

**Background:**

Cysteamine was coated to cover its odor and maintain the stability. However, coated cysteamine (CC) has not been clearly evaluated for its effects on the gastrointestinal mucosa status. We hypothesize that the appropriate CC supplementation in diet impacts the stomach and intestinal mucosa variously through regulating the morphology, apoptosis, and oxidative stress status in model of pigs.

**Results:**

The results showed that villus height increased (*P* < 0.05), and crypt depth decreased (*P* < 0.05) in the ileum when pigs were fed the diet with low cysteamine (LCS) compared with the control diet. The ileal lesion score in the LCS group was significantly (*P* < 0.01) lower than that in the control group, while the gastric lesion score in the CC group was significantly (*P* < 0.01) higher compared with that of the control group. It also showed that the activities of total superoxide dismutase (T-SOD) and diamine oxidase (DAO) were upregulated (*P* < 0.05) in the LCS group. In addition, Bax and caspase 3 immunore-activity increased (*P* < 0.01), and Bcl-2 immunoreactivity decreased (*P* < 0.01) in the gastric mucosa of pigs fed the diet with high cysteamine (HCS). The Bax and caspase 3 immunoreactivity decreased (*P* < 0.01), and Bcl-2 immunoreactivity increased (*P* < 0.01) in ileum mucosa of pigs fed the HCS diet.

**Conclusions:**

Although moderate dietary coated cysteamine showed positive effects on GI mucosal morphology, apoptosis, and oxidative stress status, the excess coated cysteamine may cause apoptosis leading to GI damage in pigs.

## Background

Cysteamine is used in oral therapy to treat cystinosis, which is an autosomal recessive lysosomal storage disease caused by mutations in the CTNS, which is the gene encoding the protein cystinosin in human [1]. Being depredated from cysteine, cysteamine is the simplest stable aminothiol. It is used as the hydrochloride salt, because it is readily oxidized to the disulphides in the presence of air. Cysteamine also improves growth of children with cystinosis [2, 3]. As a feed additive, it shows stable improvement in protein digestion and meat colour in finishing pigs [4–6]. It decreases protein breakdown, regulates hormone secretion, and reduces back fat.

Oral formulations of cysteamine may cause adverse effects, including the reduction of motor activity and generalized haemorrhage in the gastrointestinal tract and kidneys. An oral dose of 660 mg kg^− 1^ cysteamine bitartrate was fatal to rats. We found a low dose of dietary inclusion of cysteamine (at 30 mg kg^− 1^) improved growth performance and carcass quality [4]. However, the stomach fluid pH value was decreased to 2.8 (at 50 mg kg^− 1^) from 3.8 without cysteamine supplement. Oral cysteamine results in increasing gastrin and gastric acid production in laboratory animals [7–9]. Children with cystinosis and oral cysteamine administration were found to have a three-fold increase in gastric acid production and a 50% rise in serum gastrin levels above baseline [10, 11]. Thus, gastrointestinal (GI) symptoms are common in the animal receiving long term oral cysteamine. On this account, cysteamine is used to induce intestinal ulceration in an animal model. In addition to mucosal injury, a high dose (> 300 mg/kg) in a single subcutaneous injection may induce hypotension and neurological symptoms [12].

An enteric coating is a useful strategy for the oral medication or feed additive that prevents its dissolution or disintegration in the acidic environment of the stomach [13]. Ten years ago, the enteric coated cysteamine was firstly reported to reduce daily administration for patients with cystinosis [14]. However, the effect of enteric coated cysteamine on the gastrointestinal mucosa and the optimum additive range has not been systematically evaluated. Therefore, the current study aims to quantify the adverse effects of dietary CC on morphology, apoptosis, and oxidative stress status of the gastrointestinal tract in the animal model of young pigs. The results of this study may further elucidate the role of the gut on the systemic bioavailability of dietary coated cysteamine in young pigs.

## Results

### Mucosal MDA, T-SOD, T-AOC and DAO activity

**Fig. 1:**
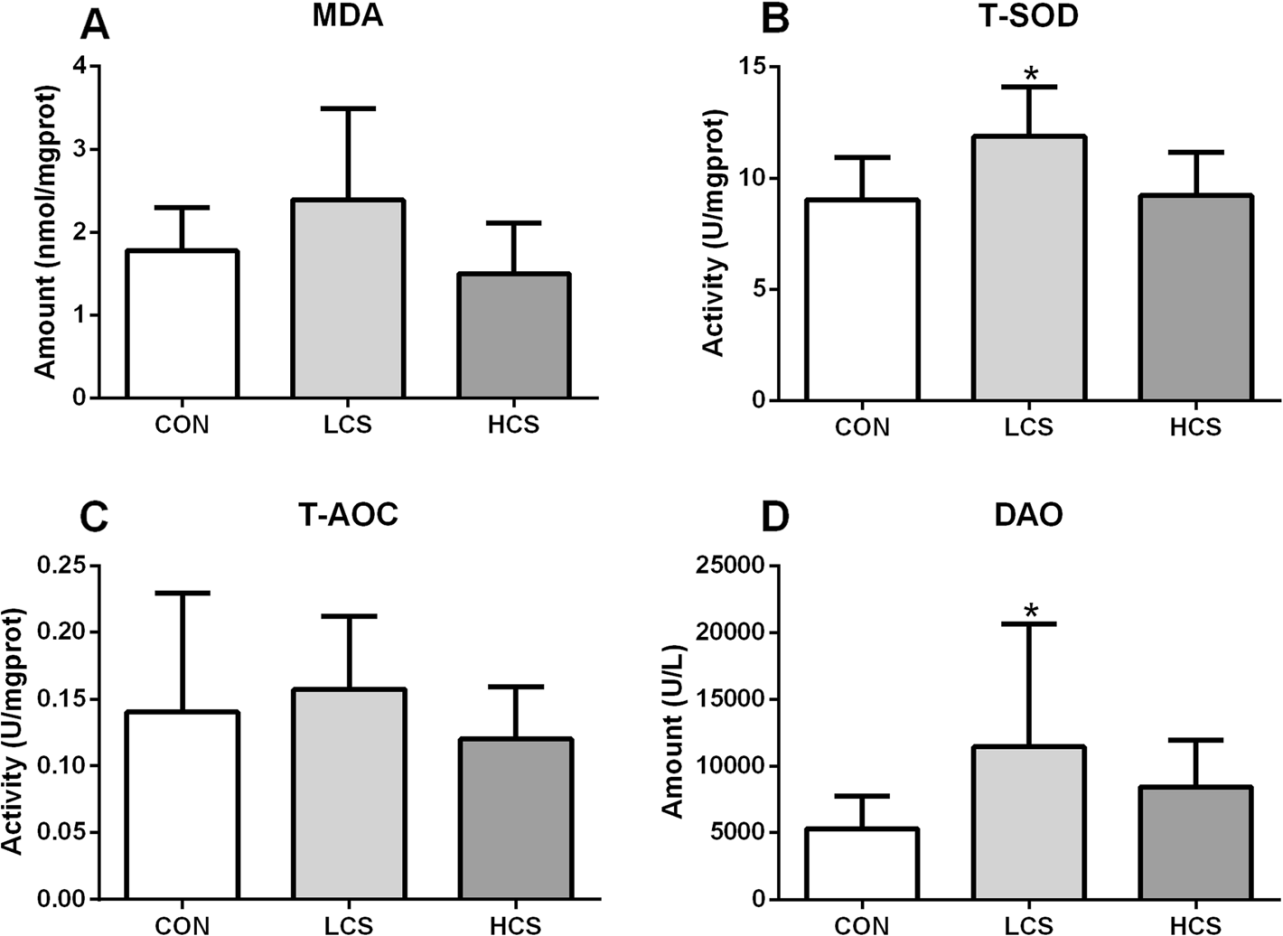
Ileum mucosal MDA (**a**), T-SOD (**b**), T-AOC (**c**) and DAO (**d**) activity. CON = pigs in the negative control group were fed a basal diet; LCS and HCS = pigs in coated cysteamine group were fed a basal diet supplemented with 35 and 280 mg/kg of cysteamine. * presents significant changes compared with CON group. Values are mean ± SEM, *n* = 8

The LCS diet elevated the activity of ileum mucosal T-SOD compared to that of the control group (*P* < 0.05) (Fig. 1b). No significant difference was found in T-SOD activity between the HCS and control group. The activity of ileum mucosal DAO was also increased in the LCS group, but not that of HCS (*P* < 0.05), in comparison with that of the control group (Fig. 1d). However, dietary coated cysteamine showed no effects on mucosal T-AOC and MDA concentrations among the groups.

### GI morphology and lesions

GI morphology in ileal was determined to evaluate the effects caused by dietary cysteamine (Table 1). Supplementation of low coated cysteamine increased ileal villus height and reduced the crypt depth (*P* < 0.05). Dietary high coated cysteamine also reduced the ileal crypt (*P* < 0.05). Villus height:crypt (V/C) ratio was increased by LCS diet compared to that of the control diet (*P* < 0.05).

**Table 1:** Effects of dietary supplementation with coated cysteamine on GI morphology and mucous damage score of finishing pigs^a^

Index	CON	LCS	HCS	SEM	*P*-value
Villus height, μm	504.1^a^	559.1^b^	522.9^ab^	14.71	0.038
Crypt depth, μm	162.7^a^	147.5^b^	143.3^b^	5.058	0.025
Villus height:crypt depth, μm:μm	3.15^a^	3.91^b^	3.72^ab^	0.153	0.003
Ileal lesion score	1.94^a^	1.00^b^	2.06^a^	0.179	< 0.001
Gastric Lesion score	1.313^a^	1.450^a^	2.125^b^	0.174	0.008
Goblet cells /100 enterocytes	10.30	8.900	8.950	0.541	0.126
Intraepithelial lymphocytes /100 enterocytes	34.65	36.25	36.25	0.702	0.199
Mucosal mast cell^b^	2.75	2.95	2.45	0.512	0.788
Submucosal mast cell^b^	8.10^a^	8.55^a^	6.50^b^	0.386	0.001
Peyer patch^c^	1.625	0.625	0.125	0.435	0.141

^a^Eight piglets per treatment. CON = pigs in the negative control group were fed a basal diet; LCS and HCS = pigs in coated cysteamine group were fed a basal diet supplemented with 35 and 280 mg/kg of cysteamine

^b^All of the cells were counted in 41,023 μm^^2^ area by microscope

^c^All of the cells were counted under 40 × 10 by microscope

**Fig. 2:**
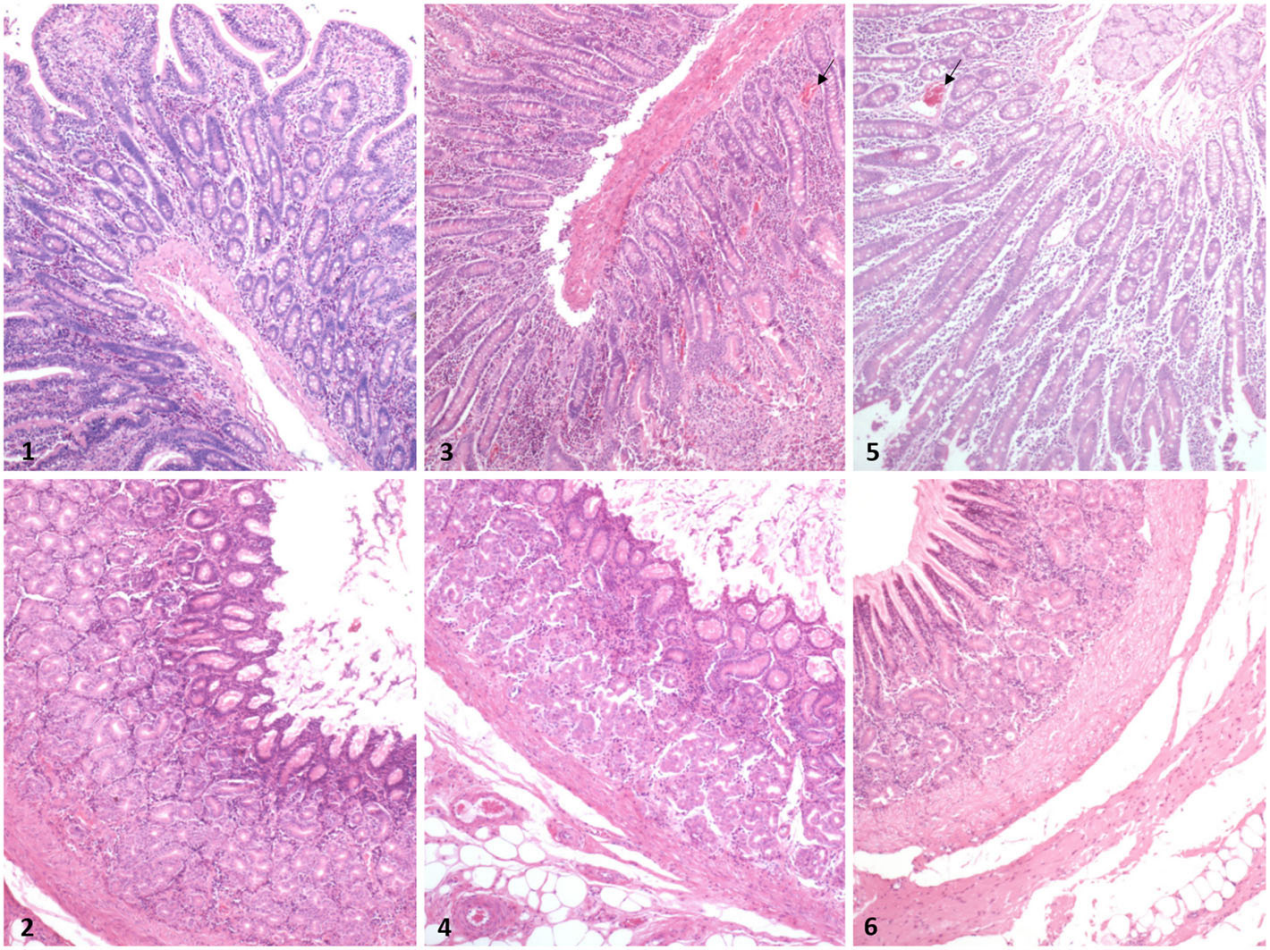
Effects of dietary supplementation with coated cysteamine on gastric and ileal morphology (HE × 40) in finishing pigs. Pigs fed a corn-soybean powder diet containing 0 (control, panels 1 and 2), 35 (LCS, panels 3 and 4) and 280 (HCS, panels 5 and 6) mg/kg of coated cysteamine hydrochloride for 29 days. There was no histological damage in the gastropore and small intestine of the control pigs. In the LCS and HCS group, Transmural hemorrhage and inflammation were seen in the ileum (arrow)

To explore the effects of coated cysteamine on pigs’ intestinal membrane barrier, we observed intestine pathologic sections and determined the intestinal mucous damage score (Table 1). The HCS group had a higher ileal lesion score compared to the control group (*P* < 0.05), whereas LCS had lower one (*P* < 0.05). In the stomach, the lesion score was increased in the HCS group (*P* < 0.05). No histological damage was found in the GI tissue of the control pigs (Fig. 2). Cell counting on the intestine pathologic section showed no effects on the amounts of intraepithelial lymphocytes, mucosal mast cells, and peyer patches induced by dietary coated cysteamine. The number of submucosal mast cells was significantly increased by the HCS diet (*P* < 0.05).

### Apoptotic marker expression by ileum compartment

**Fig. 3:**
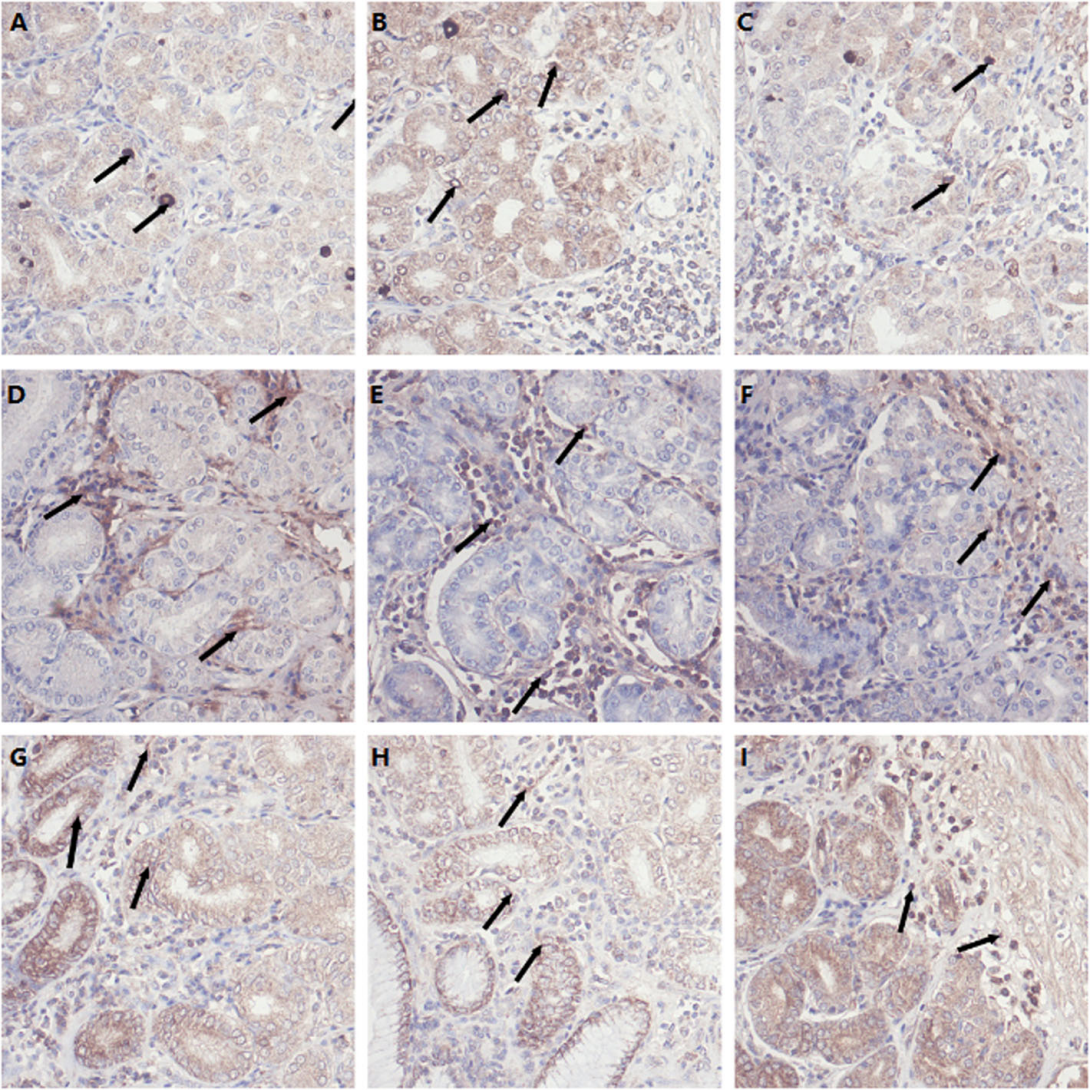
Immunohistochemical (IHC) staining identifying cells expressing anti-apoptotic Bcl-2 (**a-c**) and the pro-apoptotic Casp3 (**d-f**) and Bax (**g-i**) proteins in gastric mucosa. Positive cells are stained red-brown. Gastric mucosa of pig fed CON diet (**a**), gastric mucosa of pig fed LCS diet (**b**) and gastric mucosa of pig fed HCS diet (**c**) showing Bcl-2 cells; gastric mucosa of pig fed CON diet (**d**), gastric mucosa of pig fed LCS diet (**e**) and gastric mucosa of pig fed HCS diet (**f**) showing Casp3 cells; gastric mucosa of pig fed CON diet (**g**), gastric mucosa of pig fed LCS diet (H) and gastric mucosa of pig fed HCS diet (**i**) showing Bax cells. Arrows presented the positive protein in the mucosa

**Fig. 4:**
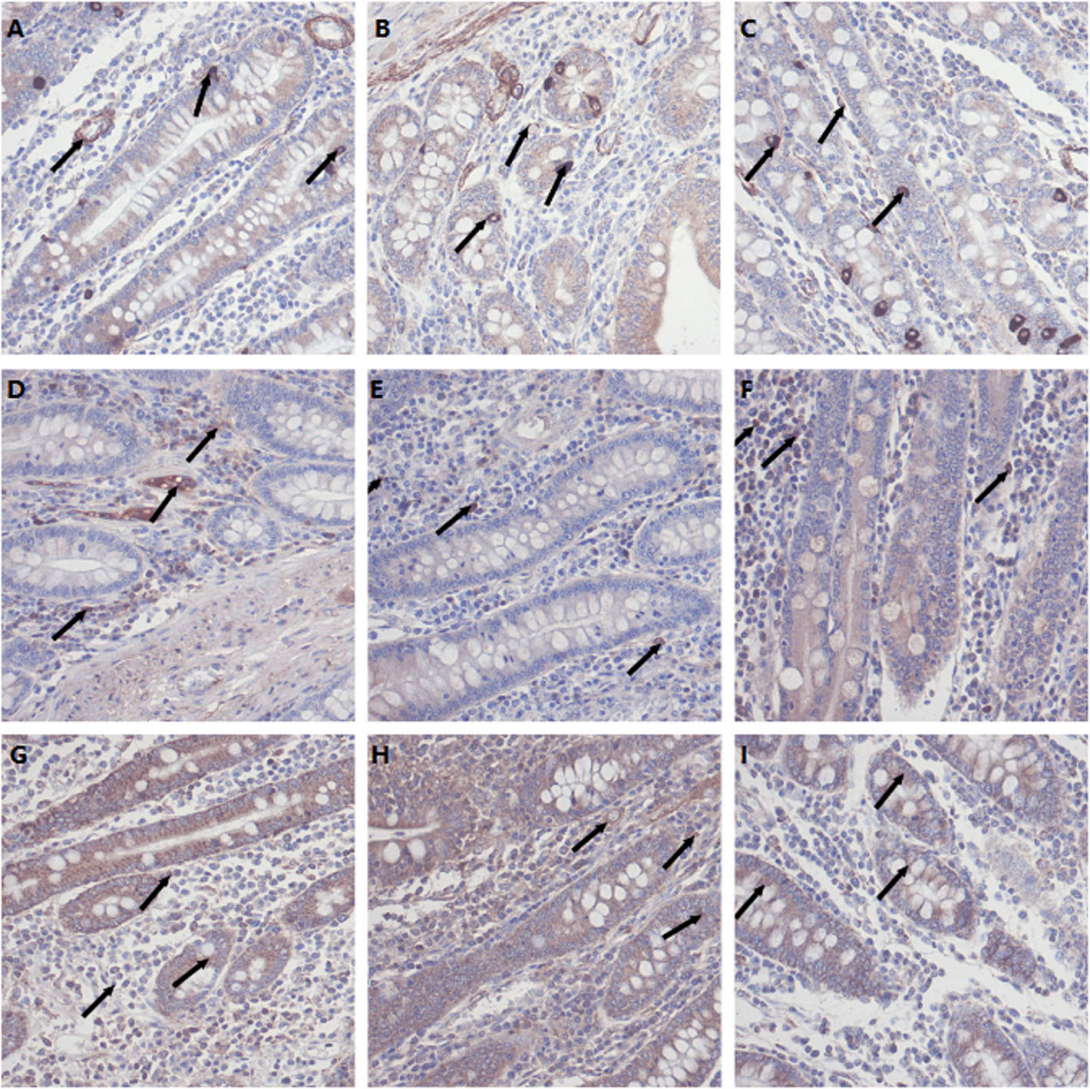
Immunohistochemical (IHC) staining identifying cells expressing anti-apoptotic Bcl-2 (**a-c**) and the pro-apoptotic Casp3 (**d-f**) and Bax (**g-i**) proteins in ilium mucosa. Positive cells are stained red-brown. Ilium mucosa mucosa of pig fed CON diet (**a**), ilium mucosa mucosa of pig fed LCS diet (**b**) and ilium mucosa of pig fed HCS diet (**c**) showing Bcl-2 cells; ilium mucosa of pig fed CON diet (**d**), ilium mucosa of pig fed LCS diet (**e**) and ilium mucosa of pig fed HCS diet (**f**) showing Casp3 cells; ilium mucosa of pig fed CON diet (**g**), ilium mucosa of pig fed LCS diet (**h**) and ilium mucosa of pig fed HCS diet (**i**) showing Bax cells. Arrows presented the positive protein in the mucosa

Apoptotic marker expression in the ileal tissue showed that bax immunoreactivity in the ileum basilar villus was reduced in the LCS group and increased in the HCS group compared to that of the control group (*P* < 0.05, Table 2). Caspase 3 immunoreactivity was increased (*P* < 0.05), and bcl-2 immunoreactivity was decreased (*P* < 0.05) in both the apical and basilar villus of pigs fed the HCS diet, whereas, the LCS diet reduced Caspase 3 immunoreactivity and elevated the bcl-2 immunoreactivity (*P* < 0.05). Immunohistochemical (IHC) staining to identify cells expressing anti-apoptotic bcl-2, pro-apoptotic caspase 3, and bax proteins in the gastric and ileum mucosa showed similar trends (Figs. 3 and 4).

**Table 2:** Apoptotic marker expression by intestinal compartment^a^

Marker, by group	Ileum apical villus	Ileum basilar villus
Bax		
CON	11,349 ± 897^ab^	2276 ± 211^a^
LCS	8603 ± 727^a^	1522 ± 157^b^
HCS	14,982 ± 1029^b^	4372 ± 348^c^
Bcl-2		
CON	8539 ± 1042^a^	2520 ± 253^a^
LCS	11,653 ± 1532^a^	4035 ± 525^b^
HCS	5186 ± 707^b^	1243 ± 142^c^
Caspase 3		
CON	7373 ± 619^a^	3037 ± 326^a^
LCS	4152 ± 409^b^	2334 ± 202^b^
HCS	11,328 ± 1066^c^	4845 ± 480^c^

^a^Eight piglets per treatment. CON = pigs in the negative control group were fed a basal diet; LCS and HCS = pigs in coated cysteamine group were fed a basal diet supplemented with 35 and 280 mg/kg of cysteamine

### Mucosal protein, DNA and RNA contents in Ileal mucous

In comparison with the control group, dietary supplementation of coated cysteamine had no effects on DNA concentrations, or the RNA/DNA or protein/DNA ratios in the ileum (Table 3).

**Table 3:** Effects of coated cysteamine supplementation on jejunum mucosal protein, DNA and RNA contents of finishing pigs^a^

Index	CON	LCS	HCS	SEM	*P*-value
Protein, mg/g tissue	3.381	3.282	3.104	0.2300	0.8934
RNA/DNA	1.978	1.904	0.7852	0.3615	0.3015
Protein/DNA, mg/μg	0.0122	0.0072	0.0046	0.0017	0.1424

^a^Eight piglets per treatment. CON = pigs in the negative control group were fed a basal diet; LCS and HCS = pigs in coated cysteamine group were fed a basal diet supplemented with 35 and 280 mg/kg of cysteamine

## Discussion

Cysteamine is formed by the decarboxylation of cysteine and the component of Coenzyme A. It is endogenously synthesized, metabolized and excreted rapidly by the animal organism [15]. Its use was approved as a dietary supplement by the FDA in 2004. In animal husbandry, cysteamine is used as a feed additive in recent years. EMEA (The European Agency for the Evaluation of Medicinal Products) summarized the oral LD_50_ value of cysteamine hydrochloride in mice is 625 mg/kg ~ 1352 mg/kg [16]. In vivo, it is quickly absorbed from the intestinal tract, and metabolized to cystamine, cysteine, glutathione and taurine. The reactivity of the compound is due to the SH and the NH2 group in the molecule. Because of its ability to produce somatostatin in the rat stomach, cysteamine has been used as a drug for the establishment of animal models of acute intestinal ulcers [17]. Plus, the characteristics such as deliquescence and pungent odors smell has to be modified by coating. However, differences in the control of the coating process and quality made the differences on the effects of supplemented coated cysteamines on the gastrointestinal mucosa.

The villi of intestine gradually became lower eventually forming a villous surface by subcutaneous or oral administration of cysteamine at 280 mg•kg^− 1^ BW [18]. Cysteamine (100 mg•kg^− 1^ bw) induced duodenal epithelial cell damage, marked exfoliation from the villous tip, was also observed at 1 h in rats after administration [19]. In this experiment, supplemented coated cysteamine at 35 mg•kg^− 1^ improved the villus height and V:C, decreased crypt depth. It is consisted with the previous report, which showed cysteamine improved intestinal integrity [20]. Crypt lengthening is accompanied by intestinal damage when assessed histologically. HCS also improved intestinal morphology by decreasing the crypt depth, which could be attributed to the protection by coating technique in present study. However, coating technology is incapable to inhibit the damage of the intestinal mucosa by high levels CC. The ileal lesion was proportional to the level of dietary cysteamine, which may be due to the thorough release of cysteamine at the ileum.

After shorten the villi, defects in the epithelium, necrosis, inflammation of the lamina propria, and a localized ulcer developed in the gastric ulcer model administrated by cysteamine [18]. GI Lesions is the indictor to monitor the GI damage. The damage in intestine and stomach was attributed to the secretion of gastric acid promoted by cysteamine [7]. In present study, supplement coated cysteamine induced gastric damage even at 35 mg•kg^− 1^ in diet. The reducing lesion score in LCS indicated the protection of cysteamine in intestine but not stomach. We found supplement cysteamine at 50 mg•kg^− 1^ in diet damage the gastric mucosa with minor parakeratosis in the stomach in 2005 [21]. In many cases, cysteamine may have opposite effects on the same parameters, depend on the supplementation dose. Cysteamine acts as a gastric ucerogenic at dose of 100 to 300 mg•kg^− 1^, but also protects against ulcerogenic effects of other compound [16]. Although Intelligent Microcapsule Technology (IMT) was used to protect intestinal damage from cysteamine, the high level cysteamine in diet elevated the gastric lesion score, indicating the lower dose could be more suitable level of coated supplementation for pigs.

Activated caspases causes the changes in mitochondrial permeability and eventually led to apoptosis. Bcl-2 inhibited the activation of caspases in mitochondria and inhibited cell apoptosis. Bax is a cell apoptosis-promoting gene belonging to bcl-2 family. The overexpression of bax antagonizes the protection of bcl-2 and lead to apoptosis. Bcl-2 also has effects on antixidation in animals. The apoptotic marker expression results indicated that as an endogenous compound, proper level of supplemented cysteamine may protect villus from apoptosis in the ileum, whereas dietary cysteamine may cause disturbance of physiological processes at abnormally high concentrations.

Coated cysteamine supplemented in pig diet could improve antioxidant status and delay meat discoloration by improving glutathione levels and antioxidase activity [22]. In mice, it showed protection against radioactivity in skin, vessels and the brain parenchyma (at around intraperitoneal doses of 250 mg•kg^− 1^ bw) [23]. The mechanism for the protection was by the antioxidant synthesis such as glutathione [24]. In this study, T-SOD and DAO activity but not glutathione (data not showed), were elevated by supplement coated cysteamine at level of 35 mg•kg^− 1^ diet, which is not in conformity with the previous reports. In rats, injected cysteamine inhibited DAO activity on two successive days at the dose of more than 400 mg•kg^− 1^ bw, indicating that high cysteamine induced ulceration, which reduced the enzyme activity [25]. In vitro, cysteamine administration increased manganese superoxide dismutase (MnSOD) activity in cultured astroglia and protected these cells from subsequent H_2_O_2_ and mechanoenzymatic stress [26]. In addition, cysteamine was about 5 times as active (in terms of increased O_2_ consumption at pH 7.5) as the previously reported peroxidase-oxidase substrates NADPH, dihydroxyfumaric acid and indol-3-ylacetic acid in vitro [27]. The disparity in results may be explained by the differences in the animal organ, intake approach (including coated or not), dose and duration.

## Conclusion

Taking together, supplemented coated cysteamine at 35 mg•kg^− 1^ diet improved the ileum mucous healthy through regulating the oxidation status and expression of apoptosis-related proteins in pigs. Although IMT helped cysteamine to promote the intestine morphology such as reducing crypt depth, excess supplemented coated cysteamine in diet may cause damage on gastrointestinal mucous.

## Methods

### Animals, experimental design, and diets

The experimental protocol was approved by the Animal Welfare Committee of the Institute of Subtropical Agriculture, The Chinese Academy of Sciences. A total of 216 crossbred finishing pigs (Duroc × Landrace × Yorkshire) with an initial BW of 88.3 ± 0.3 kg provided by a commercial farm (Jiahe biotechnology co. LTD, Hunan, Changsha, China) was randomly assigned into 3 dietary groups, fed a corn-soybean powder diet containing 0 (control), 35 (LCS), and 280 (HCS) mg•kg^− 1^ of cysteamine supplied as coated-cysteamine hydrochloride in diet for 29 days. The control diet met the nutrient requirements for finishing pigs recommended by the National Research Council (2012) and feed intakes were not affected by treatments [22]. In each group, pigs were housed in 8 pens (replicate) with 9 pigs/pen. The experiment was performed in a commercial farm (New Hope Liuhe Limited Liability Company, Yishui, China). All the animals had free access to feeds and drinking water. The basal diet met or exceeded nutrient requirements for pigs recommended by the NRC (2012) in the previous study [22]. Coated cysteamine hydrochloride, supplied by Hangzhou King Techina Technology Co., Ltd. (Hangzhou, China), contained 27% cysteamine hydrochloride. At the end of the experiment, one pig was randomly selected from each pen. The person who selected the pigs was blinded to all of the groups the pigs belonged in. Other animals were left to the farm. The selected pigs were killed by exsanguination after electrical stunning in the morning. After exsanguination, the stomach and small intestinal sample was collected. Approximately 20 cm of tissue was removed from the centre of the jejunum and ileum section [28]. Approximately 5 g intestinal tissue were flushed with ice-cold saline to recover mucosa. The mucosa samples were snap-frozen in liquid nitrogen and stored at − 80 °C. Extra intestinal samples (2 cm) and stomach (2 g) samples were rinsed with pre-cooling saline and fixed in formaldehyde solution for histological measurements [29].

#### Analysis of intestinal mucosal protein, DNA and RNA contents

Protein content of intestinal mucosal supernatant was analyzed by the method of Lowry et al. [30]. Mucosal DNA content was measured by a fluorometric assay [31]. RNA was determined by spectrophotometry by a modified Schmidt-Tannhauser method [32].

#### Mucosal MDA, T-SOD, T-AOC and DAO activity

Mucosal activities of Malondialdehyde (MDA), Superoxide Dismutase (SOD), and total antioxidative capacity (T-AOC) were measured using spectrophotometric kits in accordance with the manufacturer’s instructions (Nanjing Jiangcheng Biotechnology Institute, China). Diamine oxidase (DAO) was measured according to a previous report [33, 34].

### Evaluation of gastric and ileal mucosal lesions

To estimate the severity of gastric and ileal erosions induced by CC, gastric, and intestinal damage was scored macroscopically on a scale of 0–3 based on the severity of hyperaemia and haemorrhagic erosions as previously described: 0, almost normal mucosa; 1, mildhyperemia; 2, moderate hyperaemia and several erosions; and 3, severe hyperaemia and multiple erosions. Scoring was conducted by a person blind to treatment assignment [35].

### Determination of gastric and Ileal morphology

Haematoxylin and eosin (H & E) histological staining was performed on parallel sections of ileal tissue. Ileal tissue was immersed in 4% paraformaldehyde for 4 h and transferred to 70% ethanol. Individual lobes of ileal tissue biopsy material were placed in processing cassettes, dehydrated through a serial of alcohol gradient, and embedded in paraffin wax blocks. Ileal tissue was cut as 5 μm sections and dewaxed in xylene, rehydrated through decreasing concentrations of ethanol, washed in PBS, and then stained with haematoxylin and eosin. After staining, sections were dehydrated through increasing concentrations of ethanol and xylene. All specimens were examined under a light microscope (Nikon, Japan). Villus height and crypt depth were measured using an image-analysis system [36].

### Detection of cells positive for bcl-2, bax, and caspase-3 protein in gastrointestinal tract by immunohistochemistry

The immunohistochemical staining was performed as described by Wu et al. [37]. Bcl-2, Bax, and Caspase-3 protein were localized in the gastrointestinal tract by immunohistochemistry as previously described [38]. The antibodies used were Anti-Bax (BA0315), Anti-Bcl-2 (BA0412), Anti-Caspase-3 (BA0588), and goat anti-mouse IgG (ZSGB-BIO SP Kit).

The bcl-2, bax, and Caspase-3 positive cells were counted using a computer-supported imaging system connected to a light microscope (OlympusAX70) with an objective magnification of × 400. Then, the Bcl-2, Bax, and Caspase-3 protein expression were quantified by Image-Pro Plus 5.1 (USA) image analysis software. Each group was measured in five sections and each section was measured in five fields and averaged.

### Statistical analysis

All data were expressed as means and analyzed statistically by the one-way ANOVA using SPSS 20 (SPSS Inc., Chicago, IL, USA). Each pig was considered an experimental unit. Duncan’s multiple-range test was performed to identify differences between significant mean values. The differences were declared significant at *P* < 0.05 and a trend at 0.05 < *P* < 0.10 in all analyses, unless otherwise stated.

## Data Availability

The datasets generated and/or analysed during the current study are not publicly available to preserve the individual pigs’ and their owners’ privacy, but are available from the corresponding author on reasonable request.

## Abbreviations

bax: Bcl-2-associated X protein
bcl-2: B-cell lymphoma-2
caspase: cysteine-aspartic proteases
CC: Coat cysteamine
DAO: Diamine oxidase
GI: Gastrointestinal
IMT: Intelligent Microcapsule Technology
MDA: Malondialdehyde
MnSOD: Manganese superoxide dismutase
SOD: Superoxide Dismutase
T-AOC: Total antioxidative capacity
